# Detailed association between red blood cell distribution width and heart failure

**DOI:** 10.1002/clc.24023

**Published:** 2023-04-24

**Authors:** Teruhiko Imamura

**Affiliations:** ^1^ Second Department of Internal Medicine University of Toyama Toyama Japan

To the Editor,

Liang et al.^1^ demonstrated that red blood cell distribution width (RDW) independently predicted mortality in patients with heart failure in combination with N‐terminal pro‐B‐terminal natriuretic peptide (NT‐pro BNP) levels. Several concerns have been raised to improve their findings.

Anisocytosis, which is indicated by higher RDW values, might cause the progression of heart failure and incremental mortality due to erythrocyte deformability and impaired oxygen‐carrying capacity.^2^ Recently, hypoxia‐inducible factor‐prolyl hydroxylase inhibitors and sodium‐glucose cotransporter 2 inhibitors have been demonstrated to improve renal anemia.^3^ These medications might affect clinical outcomes including RDW levels in the heart failure cohorts. Were these medications administered to some of the participants?

Heart failure itself also has a considerable impact on the increase in RDW levels.^2^ Heart failure‐related chronic inflammation impairs iron metabolism and bone marrow function, both of which would increase RDW levels. Thus, heart failure and RDW levels have a deep association. Could the authors explain the reason why plasma NT‐pro BNP levels and RDW were “independently” associated with clinical outcomes despite their deep association? A baseline comparison between those with high RDW and those with low RDW among those with elevated plasma NT‐pro BNP might approach the mechanism. Such a comparison might clarify some therapeutic targets to improve RDW levels and clinical outcomes.

## CONFLICT OF INTEREST STATEMENT

The author declares no conflict of interest.
